# Editorial to: Where is the gap after a 90 W/4 s very high‐power short‐duration ablation of atrial fibrillation?: Association with the left atrial‐pulmonary vein voltage and wall thickness

**DOI:** 10.1002/joa3.13019

**Published:** 2024-03-15

**Authors:** Christian‐Hendrik Heeger, Karl‐Heinz Kuck, Roland Richard Tilz

**Affiliations:** ^1^ Department of Rhythmology, Cardiology and Internal Medicine Asklepios Klinik Hamburg Altona Hamburg Germany; ^2^ Department of Rhythmology, University Heart Center Lübeck University Hospital Schleswig‐Holstein Lübeck Germany; ^3^ German Center for Cardiovascular Research Partner Site Hamburg/Kiel/Luebeck Lübeck Germany

We thank Hirata et al. for their important assessment and their findings on acute pulmonary vein isolation (PVI) using 90 W/4 s‐based catheter ablation utilizing the QDOT MICRO™ ablation catheter (Biosense Webster, Inc. Diamond Bar, CA, USA).^1^ In general, catheter ablation for the treatment of atrial fibrillation (AF) by PVI has proven high efficacy and safety as well as promising long‐term success rates. Although single‐shot catheter systems provide high procedural success and safety they are mainly designed for PVI only and they lack the adaptability to different left atrial anatomies and different cardiac arrythmias. Recently, novel concepts for radiofrequency (RF), point‐by‐point and 3D mapping based catheter ablation by single‐tip ablation catheters have been evaluated. In this context high‐power short‐duration (HPSD) for a maximum of 50 W and very HPSD (vHPSD) ablation with a maximum of 90 W for 4 s have been introduced.^2^, ^3^ These concepts are aiming for increased procedure effectivity and shorter procedure time on the one hand and increased safety on the other hand by creating shallower but wider lesions due to simultaneously reducing conductive heating and increasing resistive heating.^3^

The QDOT MICRO™ Catheter is an open‐irrigated contact‐force sensing single‐tip catheter. It incorporates three microelectrodes and six thermocouples at its tip. Therefore, temperature‐controlled catheter ablation with automatic adjustment of power and irrigation flow is achieved which allows for effective lesion formation with increased safety. The QDOT Micro catheter also allows for conventional temperature‐controlled ablation for up to 50 W (QMODE). In the QMODE the system adjusts the irrigation flow rate and power based on the temperature measurement to stabilize the catheter tip temperature. In the QMODE+ mode (90 W/4 s) only power is adapted to adjust the target temperature. A switch between these two modes is always possible.^2^ Currently, two workflow concepts are performed: (1) The hybrid approach: vHPSD at posterior aspect and conventional RF ablation at the anterior aspect of the left atrial wall. (2) The vHPSD only approach utilizing 90 W/4 s only.^2^ Several studies proved both concepts, yet data on acute and chronic PVI durability assessments is limited. In the study by Hirata et al., a vHPSD only approach was utilized for PVI in 30 patients.^1^ The first‐pass PVI rate was 56% for patients and 76% for pulmonary veins. At least 30 min after encircling the pulmonary veins adenosine triphosphate testing was performed to detect potential pulmonary vein reconduction. Gaps of pulmonary vein reconduction were observed in 56% of patients. The pulmonary vein reconduction gaps were associated with the left atrial wall thickness, bipolar voltage, and the number of RF points but not with further ablation‐related parameters. The authors final conclusion is that the left atrial voltage and the wall thickness on the ablation line were highly associated with reconduction gaps using the vHPSD only ablation protocol.^1^

In our opinion, the relatively low first‐pass isolation rate of 76% and the high rate of pulmonary vein recondition found in this study is explained by the utilization of a relatively large inter‐lesion distance of 6 mm as suggested for ablation index guided conventional RF based catheter ablation (CLOSE protocol). However, for vHPSD ablation with its larger but more shallow lesion diameters it is crucial to perform a tighter inter‐lesion distance (very close protocol) at least at the anterior aspect of the pulmonary veins with its thicker tissue. This aspect is important and is correctly discussed by the authors themselves in the recent paper.^1^ The very close technique was recently described in the FAST AND FURIOUS PVI study^2^ and was proven by chronic PVI durability assessments in the remapping FAST AND FURIOUS Redo study^4^ as well as cardiac magnetic resonance imaging assessments by Sciacca et al.^5^ Utilizing vHPSD the lesion depth is only regulated by more overlapping lesions of 3–4 mm anteriorly and 5–6 mm posteriorly. This “very close protocol” concept allows for creating about 40% deeper lesions in the overlapping areas of thicker tissue.^4^ Interestingly most acute pulmonary vein reconduction gaps were found in areas of thick tissue which is one of the key findings of the study by Hirata et al. In our opinion either a hybrid approach (vHPSD posteriorly and conventional anteriorly) or a vHPSD only approach utilizing a tighter inter‐lesion distance at the anterior aspect (very close protocol) should be performed for PVI utilizing the QDOT MICRO™ Catheter. If those approaches are used high rates of first‐pass isolation as well as high rates of chronic PVI are achieved safely in a short procedure time.^2^, ^3^, ^4^, ^5^

## AUTHOR CONTRIBUTIONS

**Christian‐Hendrik Heeger**: Concept/design; data collection; data analysis and interpretation; drafting article. **Karl‐Heinz Kuck and Roland Richard Tilz**: Critical revision and approval.

## FUNDING INFORMATION

All authors declare no funding for this contribution.

## CONFLICT OF INTEREST STATEMENT

CHH received travel grants and research grants from Boston Scientific, LifeTech, Biosense Webster and Cardiofocus and speaker honoraria from Boston Scientific, Biosense Webster, Cardiofocus and C.T.I. GmbH, and Doctrina Med. KHK reports grants and personal fees from Abbott Vascular, Medtronic, Biosense Webster outside the submitted work. RRT is a consultant for Abbott, Boston Scientific, Biotronik, and Biosense Webster and received speaker honoraria from Biosense Webster, Medtronic, Boston Scientific, and Abbot Medical.

## Data Availability

Nondigital data supporting this study are curated at the study center of the department of Rhythmology, University Hospital Schleswig‐Holstein, Germany.

## References

[1] Hirata M , Nagashima K , Watanabe R , Wakamatsu Y , Hirata S , Kurokawa S , et al. Where is the gap after a 90 W/4 sec very‐high‐power short‐duration ablation of atrial fibrillation?: association with the left atrial‐pulmonary vein voltage and wall thickness. J Arrhythm. 2024. 10.1002/joa3.13009 PMC10995583 38586851

[2] Heeger C‐H , Sano M , Ștefan PS , Subin B , Feher M , Phan H‐L , et al. Very high‐power short‐duration ablation for pulmonary vein isolation utilizing a very‐close protocol—the FAST AND FURIOUS PVI study. Europace. 2022;25:880–888. 10.1093/europace/euac243 PMC10062369 36546582

[3] Reddy VY , Grimaldi M , Potter TD , Vijgen JM , Bulava A , Duytschaever MF , et al. Pulmonary vein isolation with very high power, short duration, temperature‐controlled lesions the QDOT‐FAST trial. JACC Clin Electrophysiol. 2019;5:778–786. 31320006 10.1016/j.jacep.2019.04.009

[4] Heeger C‐H , Subin B , Eitel C , Ștefan PS , Phan H‐L , Mamaev R , et al. Pulmonary vein isolation durability after very high‐power short‐duration ablation utilizing a very‐close protocol—The FAST AND FURIOUS Redo Study. IJC Heart Vasc. 2023;50:101325. 10.1016/j.ijcha.2023.101325 PMC10899720 38419611

[5] Sciacca V , Fink T , Körperich H , Bergau L , Guckel D , Nischik F , et al. Magnetic resonance assessment of left atrial scar formation following a novel very high‐power short‐duration workflow for atrial fibrillation ablation. Europace. 2023;25:1392–1399. 36815300 10.1093/europace/euac284 PMC10105851

